# APO-1/Fas gene: Structural and functional characteristics in systemic lupus erythematosus and other autoimmune diseases

**DOI:** 10.4103/0971-6866.60184

**Published:** 2009

**Authors:** Richa Singh, Vandana Pradhan, Manisha Patwardhan, K. Ghosh

**Affiliations:** Department of Immunobiology, National Institute of Immunohaematology, Indian Council of Medical Research, 13^th^ Floor, KEM Hospital Building, Parel, Mumbai - 400 012, India

**Keywords:** APO-1/Fas promoter, autoimmune diseases, systemic lupus erythematosus

## Abstract

Systemic lupus erythematosus (SLE) is an autoimmune disorder affecting multiple organ systems. It is characterized by the presence of autoantibodies reactive against various self-antigens. Susceptibility to SLE is found to be associated with many major histocompatibility complex (MHC) and non-MHC genes, one of which is APO-1/Fas gene, which is present on chromosome 10 in humans. The APO-1/Fas promoter contains consensus sequences for binding of several transcription factors that affect the intensity of Fas expression in cells. The mutations in the APO-1/Fas promoter are associated with risk and severity in various autoimmune diseases and other malignancies. The APO-1/Fas receptor is expressed by many cell types. Two forms of APO-1/Fas protein that are involved in regulation of apoptosis have been identified. Fas receptor-mediated apoptosis plays a physiological and pathological role in killing of infected cell targets. In this review, we have focused on APO-1/Fas gene structure, promoter variants and its association with SLE and other autoimmune diseases. Functional aspects of Fas receptor in apoptosis are also discussed.

## Introduction

Systemic lupus erythematosus (SLE) is an autoimmune disease mainly prevalent in females in the age group of 15-40 years and mediated by immune complexes, i.e. type III hypersensitivity. It is characterized by the production of antibodies against autoantigens or nucleoprotein particles containing DNA (nucleosomes), small nuclear ribonucleoprotein (snRNP), SS-A/Ro, SS-B/La, Sm, fragmented endoplasmic reticulum or ribosomes of diverse subcellular locations. SLE most often affects the heart, joints, skin, lungs, blood vessels, liver, kidneys and the nervous system. The course of the disease is unpredictable, with periods of illness (called *flares*) alternating with remissions. Origin of the disease is multigenic and involves different sets of genes in different individuals. There is a familial aspect to lupus, suggesting its inheritance, but it is not inherited in a typical Mendelian manner. Diagnosis of SLE is largely based on the clinical manifestations and autoantibody (anti-nuclear antibody [ANA] and anti-dsDNA) detection. It is treatable mainly with corticosteroids and immunosuppressants, but currently no cure is available.[1,2]

APO-1/Fas (CD95/TNFRSF6) receptor is a 45 kD type I transmembrane glycoprotein that belongs to the tumor necrosis factor (TNF) or neuronal growth factor receptor superfamily. It consists of 335 amino acids-a putative signal sequence of 16 amino acids, extracellular region of 155 amino acids composed of three cysteine-rich domains (CRD1, CRD2 and CRD3) and a transmembrane region of 19 amino acids followed by an intracellular part of 145 amino acids including 80 amino acids called the long “death-domain.” APO-1/Fas receptor plays a central role in physiological regulation of programmed cell death (apoptosis). It is also involved in inducing thymic selection and peripheral tolerance in the immune system. Mutations and polymorphisms in APO-1/Fas gene have been implicated in the pathogenesis of various malignancies and diseases of the immune system.[3-6]

### Organization of the human APO-1/Fas gene

**Figure 1:**
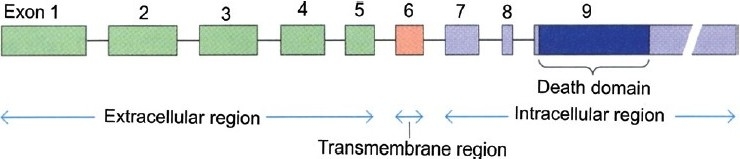
Map of the APO-1/Fas locus[9]

The human APO-1/Fas gene has been mapped to chromosome 10q24.1[7] or 10q23[8] and spans ~25 kb of the chromosome. It has nine exons (25 bp to > 1.44 kb) and eight introns (152 bp to ~12 kb). Exon 1 comprises the 5' untranslated region (UTR) and the DNA for the first 10 amino acids of the signal sequence. Exons 2-5 encode for the extracellular region and exon 6 encodes for the transmembrane region. The membrane proximal cytoplasmic 36 amino acids of the receptor are encoded by exons 7 and 8 whereas the remaining 109 amino acids, including the “death domain” and the 3' UTR having three putative polyadenylation signals, are present on exon 9 [Figure 1].[6,9]

The APO-1/Fas gene is the longest gene of its family and is highly polymorphic. APO-1/Fas gene promoter was characterized by Cheng *et al*. This 2,000-bp-long promoter is subdivided into basal promoter, enhancer region and silencer region. Consensus transcription sequences like TATA and CCAAT are absent. A GC-rich region is present upstream of the transcription start sites. It contains a high number of CpG dinucleotides and a pyrimidine-rich stretch. Consensus binding sites for transcription factors SP-1, AP-1, AP-2, GAF, NF-AT and NF-ĸB are also found in the 5' flanking sequence.[6,10,11]

### Promoter polymorphisms in systemic lupus erythematosus and other autoimmune diseases

Promoter variants in the APO-1/Fas gene have been studied in SLE and other autoimmune diseases. Single nucleotide polymorphism at nucleotide position –670 in the enhancer region, caused by A to G base change (–670 G > A), occurs at the consensus binding sequence of gamma-interferon activating site, where transcription factor signal transducer and activator of transcription 1 (STAT-1) binds and thus has an effect on the level of APO-1/Fas expression. This substitution also creates MvaI restriction fragment length polymorphism. Several studies indicate that the APO-1/Fas promoter polymorphism, 670 G > A, is associated with susceptibility to SLE. Huang *et al*. reported the association of photosensitivity and oral ulcers with the –670 AA genotype. STAT-1 binding activity was found to be higher in -670 A allele of the APO-1/Fas promoter as compared with the –670 G allele and thus may be a candidate site for SLE susceptibility. Nucleotide position –1,377 lies in between two putative silencer regions but is itself not within the sequence of consensus silencer sequence. Polymorphism –1,377 G > A affects binding of transcription factor SP-1 and hence APO-1/Fas expression. Although susceptibility to SLE is not associated with the –1,377 genotype, –670 A allele is mainly found to be linked with the –1,377 G allele. This may modulate the activity of the -670 A allele. Association of Japanese SLE patients with 297 C/416 G haplotype in APO–1/Fas gene has also been reported. [12-16]

Studies on other autoimmune diseases revealed the association of Fas –670 G > A polymorphism with disease susceptibility in multiple sclerosis (MS),[17,18] autoimmune hepatitis[19] and systemic sclerosis.[20] Contradictory results were obtained in the study of rheumatoid arthritis (RA)[14] and Sjögren's syndrome (SS).[21,22] No such association has been found in organ-specific autoimmune diseases like Hashimoto's thyroiditis,[23] Grave's disease[23] and inflammatory bowel disease.[12] It is also reported that the –1,377 A allele is associated with greater susceptibility to acute myeloid leukemia (AML) and genotype at the –670 position modulates the risk of AML only if it is associated with the –1,377 A allele and not with the –1,377 G allele.[24]

### Apoptosis and systemic lupus erythematosus

Alternative splicing of the transcript can generate two forms of the receptor-membranous Fas (mFas), anchored by the transmembrane domain and expressed on the surface of lymphocytes, epithelial cells, fibroblasts, osteoblasts and certain endothelial cells, and soluble Fas (sFas), which is the secreted form. mFas induces apoptosis when cross-linked with the Fas ligand (FasL), whereas sFas prevents cells from undergoing apoptosis.[6,25] The Fas receptor upon binding to the FasL trimerizes and induces apoptosis through the death domain that interacts with signaling adaptor Fas-associated death domain (FADD). FADD carries the death effector domain (DED) and recruits inactive procaspase 8 protein, which also has DED. The complex thus formed is called death-inducing signaling complex. Procaspase 8 is proteolytically activated to caspase 8. Activated caspase 8 cleaves and activates downstream effector caspases, including caspases 3, 6 and 7. This leads to the breakdown of several cytoskeletal and nuclear proteins and thus induces apoptosis. Caspase 3 also cleaves ICAD, the inhibitor of caspase-activated DNAse (CAD), which frees the CAD to enter the nucleus and cleave DNA. Besides the FADD/caspase 8 signaling cascade, a number of other signaling pathways also activate the Fas receptor.[26]

**Figure 2:**
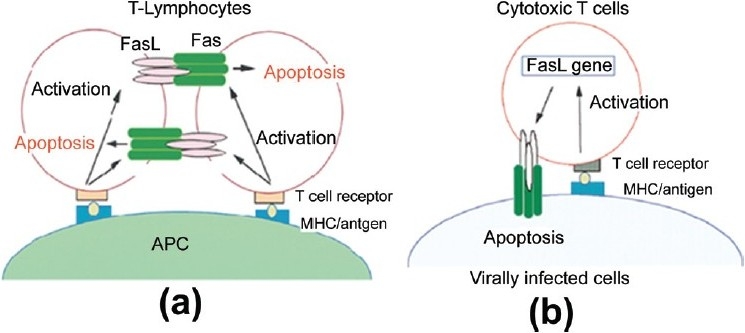
Apoptosis mediated by the Fas-FasL system[26]

FasL is expressed predominantly on *t*-lymphocytes that are activated by the interaction between *t*-Cell receptor (TCR) and antigen-presenting cells (APCs). These cells, on binding to Fas-expressing *t*-cells, lead to activation-induced cell death [Figure 2a]. Besides that, infected cells are recognized by FasL expressing cytotoxic T lymphocytes (CTLs) due to the antigen complexing with MHC. Such cells are killed by CTLs through Fas-FasL-mediated apoptosis [Figure 2b].[26]

Normally, apoptotic cells are removed by macrophages or other professional phagocytes. This regulated removal of unwanted cells by apoptosis helps in tolerance induction for autoantigens. Defects in induction of apoptosis and removal of apoptotic cells have been reported to be involved in SLE development. Deranged clearance of apoptotic cell debris leads to the release of nuclear autoantigens. These autoantigens become clustered in apoptotic blebs that are restricted to sites of free radical generation in apoptotic cells for oxidative modification. A potential source of these antigenic components is abundant generation of nucleosomes (dsDNA), which are fragments of chromatin released in cells undergoing apoptosis. These autoantigens when exposed on the surface of dying cells trigger an autoimmune response by autoreactive *t*-cells after presentation by APCs and production of autoantibodies. Immune complexes of autoantigen/autoantibody bind to the basement membrane of different organs, especially the skin and kidney, where these induce inflammation and may cause lupus nephritis, the most serious manifestation of SLE. Antibodies against dsDNA, Ro62, Ro50, La and anionic phospholipids occur commonly in SLE patients. Mutation in APO-1/Fas has been associated with the loss of regulation of B-lymphocytes, which predisposes to SLE.[27-30]

Gene upregulation precedes apoptosis. APO-1/Fas expression on cells can be induced by cytokines like IFN-γ and TNF-α.[31] Increased expression of mFas has been reported in SLE,[32] MS,[33] chronic adult *t*-cell leukemia,[34] sarcoidosis,[35] hepatitis B[36] and hepatitis C[37] infections. sFas levels in the serum are a marker for evaluating SLE disease activity. sFas is present at a high concentration in about 60% of lupus patients. Frequency of positive serum sFas is much greater with high SLE disease activity index scores than with low scores. Improvement in the clinical status of a patient after corticosteroid therapy results in decrease in sFas levels in the serum.[38,39] The levels of sFas were also found to be higher in case of scleroderma and sarcoidosis with the –670 AA, intermediate with the –670 AG and lowest with the –670 GG genotypes.[20,35] Patients with active Behcet's disease,[40] silicosis[41] and non-hematopoietic malignancies[42] also have high sFas levels.

### Other systemic lupus erythematosus susceptibility genes

Multiple factors contribute to the development of an autoimmune disease. These can be immunological, genetic, hormonal and environmental. Immunogenetic studies on autoimmune diseases have been reported from all over the world in different ethnic populations. Some polymorphisms are common risk factors for autoimmune disease development, e.g. TNF-308A variant of TNF gene at 6p21.3 for SLE, RA and primary SS and some are disease-specific, like CCR5 gene at 3p21.31 and IL1B at 2q13 for SLE.[43] Susceptibility to SLE also involves class II MHC gene polymorphism. It is found to be commonly associated with HLA-DR2 and HLA-DR3. These alleles confer two-fold to five-fold risk for lupus. Certain complement deficiencies (C1q, C2, C4a) have been strongly related to the development of SLE. Polymorphisms of genes encoding TCR, FcγR, cytokines and cytokine receptors have also been studied for their possible involvement in autoreactivity.[1]

### Future prospects

There is a need to explore genetic association in the diverse populations of India. Population studies revealed that each population holds a mutational pool in which most mutations or polymorphisms have undetectable effects on an individual, but, in combination with other alleles, it may promote or prevent from autoimmune diseases.[44] Lack of validated biomarkers for disease activity has been a barrier to drug discovery. Candidate biomarker approach is based on the study of known factors that are thought to be involved in disease pathogenesis to identify biomarkers.[45] It would be of great interest to study APO-1/Fas promoter, complement receptors, cytokines genes and their promoters and signal transducer molecules for understanding the immunopathogenesis of autoimmune diseases. This will also help to evaluate genetic and racial/ethnic influences on susceptibility, severity and treatment response in autoimmune diseases.

Protein profiling using proteomic techniques like immobilized antibody array, surface-enhanced laser desorption/ionization-time of flight-mass spectrometry and matrix-assisted laser desorption/ionization-time of flight-mass spectrometry is high throughput and does not rely on the predetermined notions of disease pathogenesis.[45,46] It has the potential of identifying novel, unexpected biomarkers that can be incorporated into multivariate prediction models. These validated proteins can then be used as input variables for drug designing. Identification of more biomarkers will definitely increase the strength of immunogenetic analysis and aid in early diagnosis of patients, severity, detecting remission and targeting the high-risk population to reduce disease acquisition.
